# Preoperative risk factors and their cumulative impact on nonsatisfaction after benign hysterectomy: A population‐based nation‐wide register study

**DOI:** 10.1111/aogs.70200

**Published:** 2026-04-08

**Authors:** Lollo Makdessi Björkström, Mats Fredrikson, Ninnie Borendal Wodlin, Lena Nilsson, Christer Borgfeldt, Preben Kjølhede

**Affiliations:** ^1^ Department of Biomedical and Clinical Sciences Linköping University Linköping Sweden; ^2^ Clinical Department of Obstetrics and Gynecology in Norrköping, Region Östergötland Norrköping Sweden; ^3^ Division of Inflammation and Infection Department of Biomedical and Clinical Sciences Linköping University Linköping Sweden; ^4^ Clinical Department of Obstetrics and Gynecology in Linköping, Region Östergötland Linköping Sweden; ^5^ Clinical Department of Anesthesiology and Intensive Care in Linköping, Region Östergötland Linköping Sweden

**Keywords:** hysterectomy, postoperative outcome, preoperative factors, risk factors, satisfaction

## Abstract

**Introduction:**

This study aimed to determine preoperative risk factors for nonsatisfaction 1 year after hysterectomy for benign indication and to analyze whether multiple co‐occurring preoperative risk factors increase the rate of nonsatisfaction.

**Material and Methods:**

A historical register study was conducted using data from the Swedish National Register for Gynecological Surgery of women aged 18–56 years, who underwent hysterectomy for benign conditions between 2004 and 2023. Satisfaction 1 year postoperatively was dichotomized into satisfaction or nonsatisfaction. Multiple logistic regression was used to evaluate preoperative risk factors, with results presented as adjusted odds ratios (aORs) and 95% confidence intervals (CIs). Nagelkerke's coefficient of determination (*R* ^2^) assessed the explanatory power of the models.

**Results:**

Among the 38 044 participating women, 3335 (8.8%) were not satisfied after 1 year. Preoperative risk factors for nonsatisfaction were smoking (aOR 1.34, 95% CI: 1.18–1.53), not being gainfully employed (aOR 1.60, 95% CI: 1.40–1.82), and the main symptoms leading to hysterectomy (pain (aOR 1.91, 95% CI: 1.70–2.15), pressure/heaviness (aOR 1.90, 95% CI: 1.53–2.21), other symptoms (aOR 2.24, 95% CI: 1.95–2.59), or several main symptoms (aOR 1.92, 95% CI: 1.53–2.41)). Protective factors for nonsatisfaction were age 46–50 years (aOR 0.87, 95% CI: 0.76–1.00) and minimally invasive hysterectomy (vaginal [aOR 0.69, 95% CI: 0.59–0.81], laparoscopic [aOR 0.79, 95% CI: 0.68–0.91], and robot‐assisted laparoscopic [aOR 0.82, 95% CI: 0.71–0.95]). The likelihood of being nonsatisfied rose with the accumulation of preoperative risk factors, as reflected by the full model's explanatory power (*R* ^2^ = 0.141). Among individual predictors, the main symptom leading to hysterectomy contributed most to the explained variance (*R* ^2^ = 0.029), followed by employment status (*R* ^2^ = 0.009), while other factors, such as surgical route, age, and smoking only marginally contributed to the variance.

**Conclusions:**

Nearly, 9% of the women were nonsatisfied with the outcome of the hysterectomy after 1 year. Lack of satisfaction appeared predictable from preoperative factors, notably smoking, not being gainfully employed, and nonbleeding symptoms leading to hysterectomy. These findings emphasize the need for individualized counseling before surgery. Given that most preoperative risk factors are resistant to immediate modification, more research is needed to develop targeted interventions that can reduce nonsatisfaction and enhance patient outcomes.

## Graphical abstract

Smoking, not being gainfully employed, and nonbleeding symptoms leading to the hysterectomy are preoperative risk factors for nonsatisfaction. Protective factors for nonsatisfaction are age 46–50 years and minimally invasive method. Individualized counseling before surgery is needed.

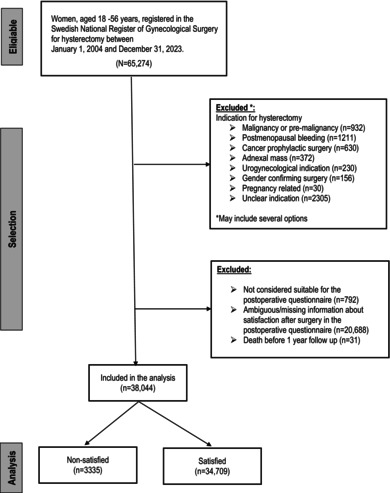

**Abbreviations**
aOR: Adjusted odds ratio
ASA: American Society of Anesthesiologists
BMI: Body mass index
CI: Confidence interval
GynOp: The Swedish National Register of Gynecological Surgery
HRQoL: Health‐related quality of life

**Key message**

A small but meaningful percentage of women who undergo a hysterectomy are not satisfied with the outcome. The number and type of certain preoperative factors may predict lack of satisfaction. These findings emphasize the need for individualized counseling before surgery.

## 1 INTRODUCTION

The main goal of hysterectomy for benign conditions is to improve health‐related quality of life (HRQoL).^1^ Many studies have focused on the surgical approach and the extent of hysterectomy,^2^ and on enhanced recovery after surgery to improve the surgical results.^3^ Enhanced recovery after surgery is a multimodal perioperative care pathway or framework to achieve early recovery for patients undergoing major surgery, and it includes preoperative preparation.^4^

More than 90% of women undergoing a benign hysterectomy are reported to be satisfied with the result, and the vast majority of patients report symptom relief and no regrets about the hysterectomy.^1^, ^5^, ^6^, ^7^, ^8^ The high satisfaction rate is seen for all surgical methods, although some differences can be seen between surgical approaches, with women preferring minimally invasive methods over abdominal hysterectomy.^2^, ^5^, ^7^, ^9^, ^10^ The level of satisfaction has been related to the cessation or relief of the symptoms that led to hysterectomy.^8^, ^11^ Women with preoperative pelvic pain, endometriosis, previous salpingitis, or mood and anxiety disorders are at higher risk of being less satisfied and of experiencing lower HRQoL after hysterectomy.^1^, ^11^, ^12^, ^13^, ^14^, ^15^, ^16^, ^17^ Indications for surgery and lifestyle factors may also affect satisfaction after surgery.^18^, ^19^

The quality of preoperative counseling, the patient's certainty about the decision to proceed with surgery, and sufficient time for the patient to reflect can also affect the patient's satisfaction after surgery.^19^, ^20^, ^21^ Women who reported being satisfied with the preoperative information were more likely to report being very satisfied after the hysterectomy.^18^, ^19^, ^20^

Therefore, the risks for patient‐experienced adverse outcomes, for example, nonsatisfaction with the result, must be addressed when deciding on surgery.^1^, ^5^, ^6^, ^7^, ^8^, ^9^ However, the risk factors for nonsatisfaction are mainly determined as individual risk factors, and whether the presence of multiple risk factors simultaneously affects the outcome regarding satisfaction with the operation has, to the best of our knowledge, not been evaluated.

To achieve a realistic understanding of the expected outcome of surgery, information about how preoperative patient‐related factors influence patient‐reported satisfaction after surgery may be important in preoperative counseling. Assessment of preoperative factors might help to identify individuals who are most likely to benefit from the procedure and those who are at risk of experiencing a less favorable outcome.^5^, ^6^, ^11^, ^12^, ^20^ However, the preoperative factors that constitute risk factors for a patient‐perceived negative outcome after hysterectomy have not been fully investigated.^18^

We hypothesize that preoperative demographics and clinical data can predict women who will not be satisfied with the result of hysterectomy 1 year after the surgery, and that the risk of nonsatisfaction increases with the number of concurrent preoperative risk factors.

The primary aim of this study was to investigate the association between preoperative patient‐related factors and the patient's experience of nonsatisfaction 1 year after hysterectomy on benign indication, and secondarily to analyze the impact of the number of preoperative risk factors on nonsatisfaction.

## 2 MATERIAL AND METHODS

This was a historical register study using prospectively collected data from the Swedish National Register of Gynecological Surgery (GynOp).^22^ The study was reported in accordance with the Strengthening the Reporting of Observational Studies in Epidemiology guidelines.^23^

The 65 274 women who were registered in the GynOp undergoing hysterectomy for benign conditions between January 1, 2004, and December 31, 2023, were eligible for the study. Inclusion criteria were age between 18 and 56 years. Exclusion criteria comprised postmenopausal women and those undergoing hysterectomy for adnexal mass, urogynecological, pregnancy‐related, cancer‐related (including prophylactic), gender‐confirming, or unclear indications. Women with histopathological evidence of malignancy or premalignancy, or predominant urogynecological symptoms, or those deemed unsuitable for postoperative questionnaires were excluded. Cases with incomplete or ambiguous satisfaction data or death within 1 year postoperatively were also excluded.

The GynOp has collected data on gynecological surgery since 1997.^22^ The register includes prospectively collected information reported in standardized forms and questionnaires by either the patient or the surgeon, or both, before the surgery, during the hospital stay, and postoperatively after 8 weeks and 12 months, respectively.

The demographic and clinical data were reported by both the woman and the doctor. The woman answered a questionnaire preoperatively about her symptoms, medical history, and lifestyle factors. She was also asked to indicate the main symptom as the reason for hysterectomy in the preoperative questionnaire by ranking six preselected categories of symptoms, in each case by writing 1 for the most important, 2 for the second most important, 3 for the third most important, etc. At the preoperative assessment, the doctor filled in a form about preoperative objective clinical findings. The surgeon who performed the hysterectomy reported information about the surgery immediately after the surgery. At discharge, the doctor filled in the postoperative form reporting the postoperative course. Eight weeks after discharge, the woman filled in a questionnaire that included questions about perceived postoperative complications. The reported complications were further assessed by the doctor. One year after the surgery, the woman answered a new questionnaire that included questions concerning satisfaction with the outcome of the surgery.

### 2.1 Data collection

Data from GynOp was obtained in May 2025.

#### 2.1.1 Dependent variables

The patient‐reported satisfaction, specified in the questionnaire on an ordinal scale as very satisfied, satisfied, neither satisfied nor dissatisfied, dissatisfied, or very dissatisfied, was dichotomized as *satisfied* when reported as very satisfied or satisfied, and as *not being satisfied* when reported as neither satisfied nor dissatisfied, dissatisfied, or very dissatisfied.

#### 2.1.2 Independent variables

The independent covariates and factors were divided into explanatory and controlling variables.

The explanatory preoperative variables consisted of age subdivided into five categories: <35 years, 36–40 years, 41–45 years, 46–50 years, and >50 years, body mass index (BMI) subdivided into three categories: normal weight (<25 kg/m^2^), overweight (25–29 kg/m^2^), and obese (≥30 kg/m^2^), parity (nulliparous or parous), smoking habits (smoker or nonsmoker), employment (gainfully employed, yes or no), sick leave status prior to hysterectomy (not on sick leave, on sick leave due to the symptoms causing the hysterectomy, or on sick leave due to other reasons), the American Society of Anesthesiologist’ (ASA) classification of physical status (ASA classes I, II, or III), a previous diagnosis of endometriosis (yes or no), previous cesarean delivery (yes or no), preoperative estrogen treatment for climacteric symptoms (yes or no), and route of hysterectomy (abdominal, vaginal, laparoscopic, or robotic‐assisted laparoscopic). The main symptom leading to hysterectomy was classified into five categories: bleeding disorder, pain conditions, lower abdominal pressure or heaviness, other symptoms, and multiple main symptoms. The symptom ranked as most important by the woman (ranked 1) was designated as the main symptom. If more than one symptom was ranked as most important, the case was categorized under multiple main symptoms. Women who had urogynecological symptoms (prolapse or urinary symptoms) as the most important symptom were excluded from the study.

The controlling variables comprised residual ovary(s) after hysterectomy (yes or no), doctor‐reported intraoperative and postoperative complications during hospital stay (yes or no), doctor‐reported complications within 8 weeks of discharge from surgery, estrogen therapy 1 year after hysterectomy (yes or no), region in Sweden where hysterectomy was performed (21 regions), and calendar year of hysterectomy categorized into four periods: 2004–2009, 2010–2014, 2015–2019, 2020–2023. Information about complications within 8 weeks of discharge was assessed by the doctor and classified by occurrence and severity as none, mild, or severe complications. Severe complications included thromboembolism, injury to either the urinary bladder, ureter, bowel, or major vascular structures, fistula, bleeding volume exceeding 1000 mL, reoperation for any reason, hospitalization for more than 7 days, complication leading to persistent physical handicap, or death. Furthermore, septic postoperative infections, and any other major complications (e.g., aspiration, allergic shock, myocardial infarction, or cerebral complications) also constituted severe complications. Mild complications, consisted of all adverse events that did not have the severity of major complications, for example, lower urinary tract infections and wound complications.

### 2.2 Primary outcome measure

The primary outcome was patient‐reported satisfaction categorized as *non‐satisfied* and *satisfied* 1 year after the hysterectomy.

### 2.3 Statistical analysis

Statistical analyses were performed using the software TIBCO Statistica™, version 13.5 (TIBCO Software Inc., Palo Alto, CA, USA), and IBM SPSS® version 28 (IBM Corp., Armonk, NY, USA).

Categorical data are presented as numbers and percentages. Univariate comparisons between the dichotomized outcomes were carried out using univariate logistic regression models. A multiple logistic regression model was used to evaluate independent risk factors. The explanatory and controlling variables were entered simultaneously into the multiple logistic regression model. To evaluate potential collinearity between the variables pairwise, a correlation matrix analysis was initially performed. Since all independent variables were categorical, Kendall's tau‐b tests were performed to assess correlations between the independent variables pairwise. The Kendall's tau‐b correlation coefficients range from −1 to 1, where −1 and 1 represent a perfect association, and 0 represents no association. Values between −0.40 and 0.40 are considered to represent weak correlations.

Missing values were substantial for some variables and would have markedly reduced the analytical sample size, resulting in a loss of statistical power. The missing values were therefore replaced by using multiple imputation in the multivariable model. Thus, the multiple logistic regression models were based on full datasets including imputed values. The results of the logistic regressions are presented as crude and adjusted odds ratios based on the pooled estimate of the imputed datasets (OR and aOR, respectively) and 95% confidence intervals (CI). The level of significance was set at *p* < 0.05.

To determine which of the significant explanatory predictors was most important, Nagelkerke's coefficient of determination (*R* ^2^) was calculated from the logistic regression, where the dependent variable was patient‐reported satisfaction categorized as *not being satisfied* or *satisfied*. Nagelkerke's *R* ^2^ gives an indication of the variation in the dependent variable that can be explained by a single or a combination of independent variables. The analysis was initially conducted with all significant explanatory variables entered simultaneously in the model. Separate analyses were then performed for each of the individual explanatory and controlling variables to evaluate the magnitude of their impact on the dependent variables.

## 3 RESULTS

**FIGURE 1:**
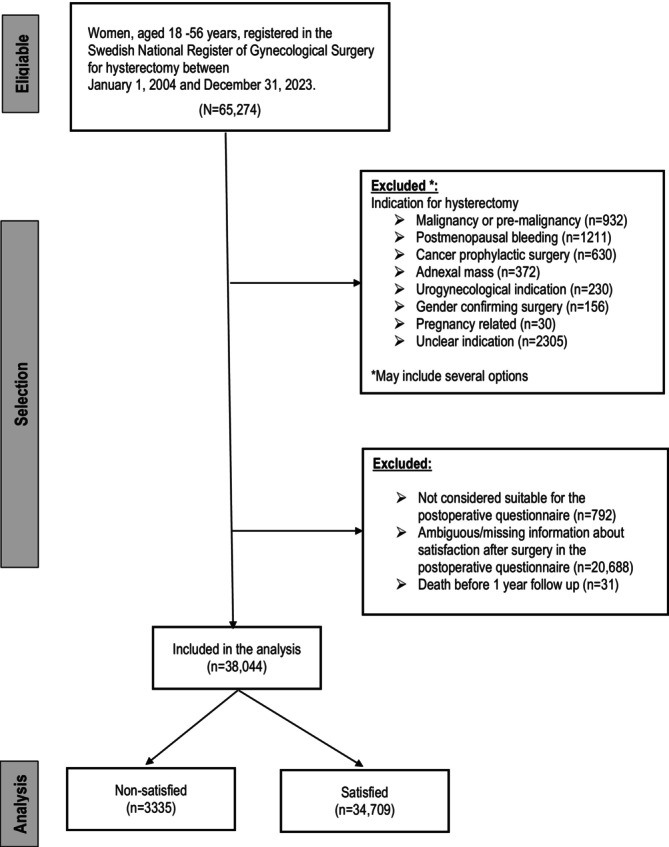
Flow chart of the study population.

The flow chart presented in Figure 1 demonstrates the selection of the study population. The final study population used for the analysis consisted of 38 044 women.

Among the 38 044 women, 3335 (8.8%) were categorized as *non‐satisfied* with the result 1 year after hysterectomy. The demographic and clinical data of the women, subdivided into *non‐satisfied* and *satisfied*, are given in Table 1. In the univariate analyses, the women who were not satisfied 1 year after hysterectomy were significantly more prevalent among women younger than 40 years of age, nulliparous women, smokers, and those with reduced physical status. A background with previous endometriosis, previous cesarean section, not being gainfully employed, and preoperative sick leave was also associated with higher proportions of nonsatisfaction. Women whose main symptom was either of the categories pain, pressure, or heaviness, other symptoms, or multiple symptoms also reported more often not being satisfied compared with women with bleeding disorders. Compared with abdominal hysterectomy, vaginal hysterectomy was associated with a lower proportion of women who were not satisfied. The absence of ovaries after surgery, complications during hospitalization, and especially severe complications within 8 weeks of discharge from surgery were strongly associated with higher proportions of women not being satisfied. In contrast, the use of estrogen therapy 1 year postoperatively correlated with a higher proportion of nonsatisfied women. Variations in the proportion of women not being satisfied were seen both regionally and temporally. Regional differences were manifested by a pair of regions showing a higher proportion of nonsatisfied women and another pair of regions showing a lower proportion. In terms of time, the categories for early years showed lower proportions of nonsatisfied women compared with the categories for later years.

**TABLE 1:** Preoperative demographic and clinical data of 38 044 women undergoing benign hysterectomy, categorized as explanatory and controlling variables, were analyzed in univariate relation to patient‐reported satisfaction 1 year after the hysterectomy.

Variable	Category	Nonsatisfied (*n* = 3335)	Satisfied (*n* = 34 709)	Univariate analysis (OR, 95%CI)
*Explanatory variables*				
Age group (years)	≤35	318 (9.5%)	2192 (6.3%)	1.56 (1.34–1.80)
	36–40	521 (15.6%)	4574 (13.2%)	1.22 (1.08–1.39)
	41–45	920 (27.6%)	9992 (28.8%)	0.99 (0.88–1.10)
	46–50	1033 (31.0%)	12 129 (34.9%)	0.91 (0.82–1.02)
	>50	543 (16.3%)	5822 (16.8%)	1.00 (reference)
BMI group	Normal	1250 (37.5%)	13 668 (39.4%)	1.00 (reference)
	Overweight	956 (28.7%)	10 666 (30.7%)	0.98 (0.90–1.07)
	Obese	602 (18.0%)	6675 (19.2%)	0.99 (0.89–1.09)
	Missing data	527 (15.8%)	3699 (10.7%)	—
Parity	Nulli parous	530 (15.9%)	4798 (13.8%)	1.26 (1.14–1.40)
	Parous	2298 (68.9%)	26 285 (75.7%)	1.00 (reference)
	Missing data	507 (15.2%)	3626 (10.5%)	—
Smoking	Nonsmoker	2398 (71.9%)	27 404 (79.0%)	1.00 (reference)
	Smoker	466 (14.0%)	4114 (11.9%)	1.29 (1.17–1.44)
	Missing data	471 (14.1%)	3191 (9.2%)	—
Gainfully employed	Yes	2274 (68.2%)	27 495 (79.2%)	1.00 (reference)
	No	579 (17.4%)	3792 (10.9%)	1.85 (1.67–2.03)
	Missing data	482 (14.4%)	3422 (9.9%)	—
On sick leave before hysterectomy	No	2115 (63.4%)	25 708 (74.1%)	1.00 (reference)
	Yes, due to symptoms causing the hysterectomy	249 (7.5%)	1960 (5.6%)	1.54 (1.34–1.77)
	Yes, for other reasons	428 (12.8%)	3094 (8.9%)	1.68 (1.51–1.88)
	Missing data	543 (16.3%)	3947 (11.4%)	—
Physical status	ASA class I	2187 (65.6%)	24 553 (70.7%)	1.00 (reference)
	ASA class II	999 (30.0%)	8774 (25.3%)	1.28 (1.18–1.38)
	ASA class III	52 (1.5%)	496 (1.4%)	1.18 (0.88–1.57)
	Missing data	97 (2.9%)	886 (2.6%)	—
Endometriosis^a^	No	2085 (62.5%)	24 526 (70.7%)	1.00 (reference)
	Yes	484 (14.5%)	3763 (10.8%)	1.51 (1.36–1.68)
	Missing data	766 (23.0%)	6420 (18.5%)	—
Cesarean section previously	No	2182 (65.4%)	24 850 (71.6%)	1.00 (reference)
	Yes	646 (19.4%)	6233 (18.0%)	1.18 (1.08–1.29)
	Missing data	507 (15.2%)	3626 (10.4%)	—
Preoperative estrogen treatment^b^	No	2678 (80.3%)	29 153 (84.0%)	1.00 (reference)
	Yes	170 (5.1%)	1660 (4.8%)	1.11 (0.95–1.31)
	Missing data	487 (14.6%)	3896 (11.2%)	—
Main symptom^c^	Bleeding disorder	1056 (31.7%)	17 938 (51.7%)	1.00 (reference)
	Pain	854 (25.6%)	6110 (17.6%)	2.37 (2.16–2.61)
	Pressure/heaviness	353 (10.6%)	3053 (8.8%)	1.96 (1.73–2.23)
	Other symptoms	423 (12.7%)	3234 (9.3%)	2.22 (1.97–2.50)
	Several main symptoms	148 (4.4%)	980 (2.8%)	2.57 (2.14–3.08)
	Missing data	501 (15.0%)	3393 (9.8%)	—
Route of hysterectomy	Abdominal	2056 (61.6%)	19 236 (55.4%)	1.00 (reference)
	Vaginal	360 (10.8%)	6234 (18–0%)	0.54 (0.48–0.61)
	Laparoscopic	458 (13.7%)	4544 (13.1%)	0.94 (0.85–1.05)
	Robot‐assisted laparoscopic	460 (13.8%)	4692 (13.5%)	0.92 (0.82–1.02)
	Missing data	1 (0.03%)	3 (0.01%)	—
*Controlling variables*				
Residual ovary(s) after hysterectomy^d^	Yes	2804 (84.1%)	30 627 (88.2%)	1.00 (reference)
	No	406 (12.2%)	2788 (8.0%)	1.59 (1.42–1.78)
	Missing data	125 (3.7%)	1294 (3.7%)	
Complications during hospital stay	No	2855 (85.6%)	31 071 (91.3%)	1.00 (reference)
	Yes	383 (11.5%)	2122 (6.1%)	2.00 (1.79–2.25)
	Missing data	97 (2.9%)	886 (2.6%)	—
Complications within 8 weeks of discharge	No	752 (22.5%)	18 339 (52.8%)	1.00 (reference)
	Mild	1129 (33.9%)	10 265 (29.6%)	2.68 (2.44–2.95)
	Severe	993 (29.8%)	3160 (9.1%)	7.66 (6.92–8.49)
	Missing data	461 (13.8%)	2945 (8.5%)	—
Estrogen therapy 1 year after hysterectomy^e^	No	2656 (79.6%)	29 649 (85.4%)	1.00 (reference)
	Yes	506 (15.2%)	3402 (9.8%)	1.66 (1.50–1.84)
	Missing data	173 (5.2%)	1658 (4.8%)	—
Region in Sweden^f^	Region Västra Götaland	779 (23.4%)	8184 (23.6%)	1.00 (reference)
	Region Stockholm	454 (13.6%)	4054 (11.7%)	1.18 (1.04–1.33)
	Region Skåne	367 (11.0%)	3900 (11.2%)	0.99 (0.87–1.13)
	Region Östergötland	218 (6.5%)	2096 (6.0%)	1.09 (0.93–1.28)
	Region Halland	153 (4.6%)	1852 (5.3%)	0.87 (0.72–1.04)
	Region Jönköping	124 (3.7%)	1565 (4.5%)	0.83 (0.68–1.01)
	Region Uppsala	175 (5.2%)	1287 (3.7%)	1.43 (1.20–1.70)
	Region Norrbotten	112 (3.4%)	1328 (3.8%)	0.89 (0.72–1.09)
	Region Dalarna	127 (3.8%)	1248 (3.6%)	1.07 (0.87–1.32)
	Region Västernorrland	87 (2.6%)	1207 (3.5%)	0.76 (0.60–0.95)
	Region Gävleborg	110 (3.3%)	1182 (3.4%)	0.98 (0.79–1.20)
	Region Västerbotten	115 (3.4%)	1126 (3.2%)	1.07 (0.87–1.32)
	Region Örebro	95 (2.8%)	1131 (3.3%)	0.88 (0.71–1.10)
	Region Kalmar	63 (1.9%)	967 (2.8%)	0.68 (0.52–0.89)
	Region Kronoberg	79 (2.4%)	933 (2.7%)	0.89 (0.70–1.13)
	Region Sörmland	78 (2.3%)	678 (2.0%)	1.21 (0.95–1.55)
	Region Jämtland	46 (1.4%)	588 (1.7%)	0.82 (0.60–1.12)
	Region Blekinge	59 (1.8%)	479 (1.4%)	1.29 (0.98–1.71)
	Region Västmanland	46 (1.4%)	417 (1.2%)	1.16 (0.85–1.59)
	Region Värmland	39 (1.2%)	422 (1.2%)	0.97 (0.69–1.36)
	Region Gotland	9 (0.3%)	65 (0.2%)	1.45 (0.72–2.93)
Year of hysterectomy	2004–2009	667 (20.0%)	8895 (25.6%)	0.69 (0.62–0.77)
	2010–2014	701 (21.0%)	7989 (23.0%)	0.81 (0.73–0.90)
	2015–2019	1075 (32.2%)	9603 (27.7%)	1.03 (0.94–1.13)
	2020–2023	892 (26.7%)	8222 (23.7%)	1.00 (reference)

*Note*: Figures denote as number and (%).

Abbreviations: ASA, American Society of Anesthesiologists; BMI, body mass index; CI, confidence interval; OR, odds ratio.

^a^ History of endometriosis prior to hysterectomy.

^b^ Systemic estrogen therapy for climacteric symptoms.

^c^ Main symptoms leading to hysterectomy.

^d^ At least one residual ovary after hysterectomy.

^e^ Systemic estrogens for climacteric symptoms or estrogen replacement therapy.

^f^ Region in Sweden where the hysterectomy was performed.

Before the multivariable analysis was conducted, an analysis of pairwise correlations between the independent variables was performed and showed that Kendall's tau‐*b* correlation coefficients ranged between −0.31 and 0.38, indicating no or weak collinearity between the variables (data not shown).

In the multivariable model (Table 2), most of the significant differences in the univariate analysis of the explanatory variables disappeared. The explanatory variables that remained independent risk factors for nonsatisfaction were smoking, not being gainfully employed, the main symptom categories pain, pressure/heaviness, other symptoms, and multiple main symptoms. In contrast, being aged 46–50 years was found to be a protective factor for not being satisfied, as were all modes of minimally invasive hysterectomy. Of the controlling variables, complications during hospital stay, complications within 8 weeks of discharge, and estrogen therapy 1 year after hysterectomy remained independent risk factors of nonsatisfaction. Only one of the two regions with a higher proportion of nonsatisfied women and one of the two regions with lower proportions remained as independent risk factors while another region was added as an independent protective factor for having a lower proportion. The proportion of nonsatisfied women did not change across the time periods.

**TABLE 2:** Multivariable associations between preoperative demographic and clinical factors and nonsatisfaction 1 year after hysterectomy among 38 044 women, with missing data handled by multiple imputation.

Variable	Category	Multiple logistic regression^a^ (aOR, 95% CI)	*p*‐value
*Explanatory variables*			
Age group (years)	≤35	0.98 (0.78–1.22)	0.82
	36–40	0.95 (0.79–1.13)	0.56
	41–45	0.94 (0.82–1.09)	0.42
	46–50	0.87 (0.76–1.00)	0.048
	>50	1.00 (reference)	
BMI group	Normal	1.00 (reference)	
	Overweight	0.94 (0.84–1.04)	0.24
	Obese	0.89 (0.79–1.01)	0.07
Parity	Nulli parous	1.01 (0.88–1.15)	0.90
	Parous	1.00 (reference)	
Smoking	Nonsmoker	1.00 (reference)	
	Smoker	1.34 (1.18–1.53)	<0.0001
Gainfully employed	Yes	1.00 (reference)	
	No	1.60 (1.40–1.82)	<0.0001
On sick leave before hysterectomy	No	1.00 (reference)	
	Yes, due to symptoms causing the hysterectomy	1.12 (0.94–1.33)	0.22
	Yes, for other reasons	1.07 (0.91–1.24)	0.42
Physical status	ASA class I	1.00 (reference)	
	ASA class II	1.07 (0.97–1.19)	0.19
	ASA class III	0.89 (0.61–1.30)	0.56
Endometriosis^b^	No	1.00 (reference)	
	Yes	0.96 (0.82–1.13)	0.65
Cesarean section previously	No	1.00 (reference)	0.45
	Yes	1.05 (0.93–1.18)	
Preoperative estrogen treatment^c^	No	1.00 (reference)	
	Yes	1.15 (0.94–1.41)	0.18
Main symptom^d^	Bleeding disorder	1.00 (reference)	
	Pain	1.91 (1.70–2.15)	<0.0001
	Pressure/heaviness	1.90 (1.53–2.21)	<0.0001
	Other symptoms	2.24 (1.95–2.59)	<0.0001
	Several main symptoms	1.92 (1.53–2.41)	<0.0001
Route of hysterectomy	Abdominal	1.00 (reference)	
	Vaginal	0.69 (0.59–0.81)	<0.0001
	Laparoscopic	0.79 (0.68–0.91)	<0.01
	Robot‐assisted laparoscopic	0.82 (0.71–0.95)	<0.01
*Controlling variables*			
Residual ovary(s) after hysterectomy^e^	Yes	1.00 (reference)	
	No	1.03 (0.87–1.21)	0.74
Complications during hospital stay	No	1.00 (reference)	
	Yes	1.39 (1.19–1.61)	<0.0001
Complications within 8 weeks of discharge	No	1.00 (reference)	
	Mild	2.44 (2.19–2.73)	<0.0001
	Severe	6.85 (6.08–7.73)	<0.0001
Estrogen therapy 1 year after hysterectomy^f^	No	1.00 (reference)	
	Yes	1.45 (1.25–1.67)	<0.0001
Region in Swedenh^g^	Region Västra Götaland	1.00 (reference)	
	Region Stockholm	0.99 (0.83–1.17)	0.88
	Region Skåne	1.00 (0.85–1.17)	0.97
	Region Östergötland	1.05 (0.87–1.28)	0.60
	Region Halland	0.86 (0.68–1.09)	0.21
	Region Jönköping	0.87 (0.68–1.11)	0.26
	Region Uppsala	1.26 (1.01–1.58)	0.04
	Region Norrbotten	0.71 (0.52–0.97)	0.03
	Region Dalarna	1.05 (0.82–1.34)	0.71
	Region Västernorrland	0.80 (0.59–1.07)	0.13
	Region Gävleborg	0.81 (0.62–1.05)	0.11
	Region Västerbotten	1.04 (0.81–1.33)	0.75
	Region Örebro	0.81 (0.60–1.09)	0.17
	Region Kalmar	0.72 (0.53–0.98)	0.03
	Region Kronoberg	0.99 (0.75–1.31)	0.97
	Region Sörmland	0.96 (0.69–1.32)	0.79
	Region Jämtland	0.84 (0.58–1.23)	0.37
	Region Blekinge	1.09 (0.75–1.58)	0.67
	Region Västmanland	1.26 (0.84–1.90)	0.27
	Region Värmland	0.90 (0.60–1.33)	0.58
	Region Gotland	1.23 (0.51–3.00)	0.65
Year of hysterectomy	2004–2009	0.88 (0.75–1.02)	0.10
	2010–2014	0.96 (0.83–1.11)	0.61
	2015–2019	1.13 (1.00–1.27)	0.050
	2020–2023	1.00 (reference)	

Abbreviations: aOR, adjusted odds ratio; ASA, American Society of Anesthesiologists; BMI, body mass index; CI, confidence interval.

^a^ All explanatory and controlling variables were entered simultaneously as confounders in the model.

^b^ History of endometriosis prior to hysterectomy.

^c^ Systemic estrogen therapy for climacteric symptoms.

^d^ Main symptoms leading to hysterectomy.

^e^ At least one residual ovary after hysterectomy.

^f^ Systemic estrogens for climacteric symptoms or estrogen replacement therapy.

^g^ Region in Sweden where the hysterectomy was performed.

The explained proportion of the variance of the significant preoperative risk factors in the multivariable logistic regression model of nonsatisfaction was 14% (*R* ^2^ = 0.141). The individual independent variables' contribution to the explained variance is given in Table 3.

**TABLE 3:** Nagelkerke's determination coefficients (*R* ^2^) from the logistic regression models assessing the explained variance of risk factors for nonsatisfaction 1 year after hysterectomy.

Independent variables	*R* ^2^
Full model including all significant independent explanatory variables	0.141
Individual explanatory preoperative variables	
Independent risk factors (listed in order of magnitude of *R* ^2^)	
Main symptom leading to hysterectomy	0.029
Gainfully employed	0.009
Route of hysterectomy	0.007
Age	0.004
Smoking	0.001
Not independent risk factors (listed in order of magnitude of *R* ^2^)	
On sick leave before hysterectomy	0.007
Endometriosis previously	0.005
Parity	0.003
Physical status	0.001
Cesarean section previously	0.001
BMI	0.0005
Preoperative estrogen treatment	0.0005
Individual independent controlling variables (listed in order of magnitude of *R* ^2^)	
Complications during hospital stay	0.007
Complications within 8 weeks after hysterectomy	0.007
Estrogen therapy 1 year after hysterectomy	0.005
Year of hysterectomy	0.005
Residual ovary(s) after hysterectomy	0.004
Region in Sweden	0.004

Among the significant explanatory preoperative risk factors, the main symptom categories leading to hysterectomy, that is, pain, pressure/heaviness, other symptoms, and multiple main symptoms, had the strongest impact on the explained variance followed by not gainfully employed, route of hysterectomy, age, and smoking, which together accounted for 35% (0.050/0.141) of the *R* ^2^ for the full model. These factors accounted for 58% (0.029/0.050), 18% (0.009/0.050), 14% (0.007/0.050), and 2% (0.001/0.050), respectively, of the explained variance, which implies that for each co‐occurring preoperative risk factor, the risk of nonsatisfaction increased incrementally but not proportionally.

The significant controlling risk factors, i.e., complications during hospital stay, complications within 8 weeks of discharge, estrogen therapy 1 year after hysterectomy, and the region in Sweden accounted together for another 16% of the explained variance. In addition, the nonsignificant explanatory and controlling factors together accounted for an additional 19% of the explained variance.

## 4 DISCUSSION

The study showed that almost one in 11 women was not satisfied with the result of a hysterectomy after 1 year. Some independent preoperative risk and risk‐reducing factors for nonsatisfaction were identified. Being a smoker, not being gainfully employed, and having a main symptom other than a bleeding disorder as an indication for hysterectomy were risk factors for nonsatisfaction. In contrast, being 46–50 years old and using minimally invasive hysterectomy methods were risk‐reducing factors for not being satisfied with the result. The risk of not being satisfied increased with the number of concurrent preoperative risk factors. Each of the risk factors contributed to varying degrees to the total variation, with the main symptom leading to hysterectomy and not being gainfully employed making the strongest contributions.

The proportion of women who were not satisfied with the result of hysterectomy in this study, and consequently also the proportion who were satisfied, was comparable to that reported by other researchers.^6^, ^7^, ^11^ The literature on patient‐reported satisfaction after hysterectomy is relatively scarce, and the concept of satisfaction after hysterectomy has been conflated with the concept of regret about the procedure. Although these two concepts may be closely related, they do not necessarily mean the same thing. While nonsatisfaction can be perceived as a signal for change, regret should be seen as a feeling of sadness over a past event that cannot be undone. In connection with this study, however, we chose to use the broader meaning of the concept of satisfaction to be able to compare broadly with the findings on satisfaction and regretting in the literature.

Several of the preoperative explanatory factors were found to be risk factors for nonsatisfaction after hysterectomy in the univariate analyses, but after adjusting for known and potential confounding factors, only smoking, not being gainfully employed, and having a main symptom category other than bleeding disorder as an indication for hysterectomy remained independent risk factors.

Smoking and not being gainfully employed may be seen as proxy measures for socioeconomic status. The cause of smoking is multifactorial and consists of physical, social, behavioral, psychological, and mental health factors.^24^ The association between smoking and unemployment has previously been established.^25^ Smoking has even been recognized as an important risk factor for postoperative complications.^26^ Moreover, studies have shown that lower socioeconomic status, including factors such as smoking and employment status, is associated with lower satisfaction after surgery.^27^, ^28^, ^29^ In our study, however, the analysis was adjusted for complications, and both smoking and complications still remained independent risk factors for nonsatisfaction, indicating that smoking itself may have a negative effect on satisfaction. Thus, our findings regarding smoking and employment as risk factors for nonsatisfaction were consistent with the existing literature.

In this study, the main symptom categories leading to hysterectomy other than bleeding disorders were also found to be independent risk factors for nonsatisfaction after surgery. All main symptom categories revealed an approximately twofold increase in the likelihood for nonsatisfaction. Only a very few publications have addressed the association between indications for hysterectomy and the subsequent experienced satisfaction. Although Kupperman et al.^11^ and Janda et al.^5^ in their large series of women undergoing hysterectomy, 1420 and 2319 women, respectively, reported the symptom category or reason for hysterectomy, they did not analyze the association between symptom category or reason for hysterectomy and patient‐reported satisfaction. However, Borendal Wodlin,^9^ presented data on the association showing that pain and uterine myomas as indications for hysterectomy were overrepresented in women who were not satisfied with their hysterectomy. Borendal Wodlin's study was, like the present study, based on data from the GynOp but from the period January 2004 to June 2016 whereas the present study's period was January 2004–December 2023. The categorization of the main indications for hysterectomy varies slightly between that study and the present study, which makes it a little more difficult to directly compare the results for all categories. However, both studies used bleeding or menstrual disorders as a reference. Pain, as well as uterine myoma (the latter equivalent to pressure/heaviness), were independent risk factors in both studies. The reasons for the symptom pressure/heaviness being a risk factor for nonsatisfaction with hysterectomy are not entirely obvious. Although hysterectomy should effectively relieve the mechanical symptoms of an enlarged uterus, for example, the grief at losing the uterus, especially if there was still a desire to have children, would be a reason to regret hysterectomy or be less satisfied.^30^ The explanation for our finding is speculative, however, but there appear to be other unknown factors than those reported in the registry data that may play a role. Preoperative pain has been associated with persistent pain and with impaired HRQoL and thus also with a reduced likelihood of satisfaction and an increased risk of regretting hysterectomy.^3^, ^9^, ^12^, ^13^, ^15^, ^16^, ^17^ Thus, our findings are consistent with the existing literature.

Women with other indications and those with multiple main symptoms constitute groups that either have more diffuse or less well‐defined symptoms or have several somatic symptoms that they describe as the main symptoms that led to hysterectomy. Such women were also at a higher risk of nonsatisfaction in our study. A possible reason for this could be that these women were probably more prone to develop a chronic course of illness and were therefore more likely to feel nonsatisfied after a hysterectomy because not all symptoms disappeared.^31^

In this study, being aged 46–50 years and the use of minimally invasive hysterectomy methods were risk‐reducing factors for nonsatisfaction 1 year after hysterectomy. Previous studies have shown conflicting results on the association between age and patient regret about hysterectomy,^30^, ^32^, ^33^ while there is strong scientific support for greater satisfaction after minimally invasive methods of hysterectomy.^5^, ^7^, ^9^, ^10^

Regarding the effect of controlling variables on satisfaction, complications of surgery constituted strong independent risk factors for nonsatisfaction, which is in line with other studies.^6^, ^9^, ^34^ This result supports the opinion that greater efforts should be made to prevent complications to reduce suffering, healthcare costs, and the resulting nonsatisfaction with the procedure afterwards. Loss of both ovaries at the time of hysterectomy did not appear to be a risk factor for nonsatisfaction. This is somewhat remarkable as loss of estrogen is associated with reduced HRQoL, and therefore loss of both ovaries would be expected to cause a greater degree of nonsatisfaction.^35^, ^36^ Previous studies have shown that although the satisfaction rate was high overall, regret persisted especially after oophorectomy.^35^ Estrogen therapy 1 year after hysterectomy should be anticipated to counteract the negative effect on satisfaction of loss of the ovaries, but it seemed that estrogen therapy instead was an independent risk factor for nonsatisfaction, probably reflecting that these women were not sufficiently relieved from climacteric symptoms and related the symptoms to hysterectomy. This emphasizes the importance of providing a sufficient dosage of estrogen to ensure adequate effect of the treatment on menopausal symptoms.

The reasons for the variations in prevalence of nonsatisfaction after hysterectomy between regions are speculative, but may reflect regional variations in sociodemographics, healthcare organization, clinical practice, and sociocultural contexts, or may simply represent an effect of a multiple testing phenomenon.

The likelihood of nonsatisfaction increased with the number of concurrent preoperative risk factors. Each of the risk factors contributed differently to the total variation, with the main symptom leading to hysterectomy and not being gainfully employed making the strongest contributions. These findings provide important new knowledge that can be applied clinically to improve preoperative counseling about hysterectomy and to help women develop realistic expectations regarding the outcome of hysterectomy. *R* ^2^ is a measure of the proportion of variance that is explained by an independent variable or a combination of variables. Although the analyses did not reveal significant interaction effects between the independent variables, the *R* ^2^ for the full model including all significant preoperative risk factors, was higher than the sum of the *R* ^2^'s for the individual explanatory and controlling variables, indicating that the independent variables might interact with each other. However, the small difference (0.012) is probably explained by Nagelkerke's *R* ^2^ not being a precise measure.

The major strength of this study was the large sample size, consisting of a nation‐wide population. In addition, GynOp is a national registry with high coverage and validated data quality, demonstrating strong internal validity.^21^, ^37^ Another strength was the use of multiple imputation for substituting missing data. Multiple imputation replaces missing values with plausible estimates derived from observed data patterns. This approach was chosen because it reduces bias and better preserves variability compared with single imputation methods. The procedure assumes that data are missing at random, an assumption that allows valid statistical inference.^38^

A limitation was that the variables in the register were restricted and therefore may not explain complex issues or fully evaluate risk factors. For instance, the register does not provide information on all lifestyle factors or on anxiety and depression symptoms, which are known factors for satisfaction after hysterectomy.^14^, ^15^, ^36^ Furthermore, some register variables have changed over time, with new variables added and others removed. Such changes may introduce bias due to missing data for certain individuals. Other shortcomings with register studies in general are the risk of selection bias and the possibility that essential information is missing from the registry.^39^ However, given the high coverage of GynOp (91–98%), these risks may be considered minimal.^37^ Thus, the generalizability of this study may be restricted to societies and healthcare environments with similar characteristics and functions as the Swedish one.

## 5 CONCLUSION

A small but not insignificant percentage of the women were not satisfied with the result of their hysterectomy 1 year after it was performed. Nonsatisfaction appeared to be predictable based on some preoperative patient‐related lifestyle, social, and clinical factors. The preoperative consultation should be adapted to the patient, and factors that can influence satisfaction should be addressed before the decision on surgery is made. However, most of these preoperative factors are unlikely to be preventable or modifiable in the short term. Further studies are warranted to investigate whether this suggested approach can reduce the proportion of nonsatisfied women after hysterectomy.

## AUTHOR CONTRIBUTIONS

LMB and PK conceptualized the study. LMB, PK, NBW, and LN participated in study planning and elaboration of the study protocol. LMB and PK processed the database from the GynOp. MF, LMB and PK conducted the statistical analyses. LMB is the primary author. PK, MF, LN, NBW and CB revised the draft. All authors approved the final version of the manuscript.

## FUNDING INFORMATION

The study was supported financially by the Department of Obstetrics and Gynecology in Norrköping, the Medical Research Council of Southeast Sweden (FORSS‐969521, recipient LMB), and by ALF grants from Region Östergötland (RÖ‐936208, recipient PK). The funding sources were not involved in the study design, collection and analysis of data, report writing, or the decision to submit for publication.

## CONFLICT OF INTEREST STATEMENT

The authors have no conflicts of interest to declare.

## ETHICS STATEMENT

The study was approved by the Swedish Ethical Review Authority (Dnr. 2024‐08505‐01, date of approval January 29, 2025). All women registered in the GynOp receive written information about the register before surgery and consent to participate in research according to Swedish legislation. In addition, the information includes a statement about the opportunity to decline or withdraw participation later if requested.

## Data Availability

The data that support the findings of this study are available on request from the corresponding author, with due approval from the relevant authority according to Swedish legislation. The data are not publicly available due to privacy or ethical restrictions.
